# Epidemiologic Characteristics, Transmission Chain, and Risk Factors of Severe Infection of COVID-19 in Tianjin, a Representative Municipality City of China

**DOI:** 10.3389/fpubh.2020.00198

**Published:** 2020-05-20

**Authors:** Jin Wang, Zhihui Li, Xiaomin Cheng, Huan Hu, Conghui Liao, Pengyuan Li, Jiahai Lu, Zeliang Chen

**Affiliations:** Department of Epidemiology, School of Public Health, Sun Yat-sen University, Guangzhou, China

**Keywords:** coronavirus disease 2019, SARS-CoV-2, epidemiologic characteristics, transmission chain, risk factors, gender

## Abstract

This study was performed to describe the epidemiologic characteristics of coronavirus disease 2019 (COVID-19) and explore risk factors for severe infection. Data of all 131 confirmed cases in Tianjin before February 20 were collected. By February 20, a total of 14/16 districts reported COVID-19 cases, with Baodi district reporting the most cases (*n* = 56). A total of 22 (16.8%) cases had a Wuhan-related exposure. Fever was the most common symptom (82.4%). The median duration of symptom onset to treatment was [1.0 (0.0–4.0) days], the duration of symptom onset to isolation [2.0 (0.0–6.0) days], and the duration of symptom onset to diagnosis [5.0 (2.0–8.0) days]. The analysis of the transmission chain showed two cluster infections with 62 cases infected. Transmission from a family member constituted 42%, usually at the end of transmission chain. Compared with patients with non-severe infections, patients with severe infections were more likely to be male (46.2 vs. 77.3%, *P* = 0.009) and had a Wuhan-related exposure (14.0 vs. 40.9%, *P* = 0.004). Multivariate logistic regression showed that male (OR 3.913, 95% CI 1.206, 12.696; *P* = 0.023) was an independent risk factor for severe infection. This study provides evidence on the epidemic of COVID-19 by analyzing the epidemiological characteristics of confirmed cases in Tianjin. Self-quarantine at an outbreak's early stage, especially for those with high-risk exposures, is conducive to prevent the transmission of infection. Further investigation is needed to confirm the risk factors for severe COVID-19 infection and investigate the mechanisms involved.

## Introduction

In December 2019, the local Centers for Disease Control and Prevention (CDC) in Wuhan City, Hubei Province, China reported a cluster of unexplained pneumonia cases. The infections, named as coronavirus disease 2019 (COVID-19) by the World Health Organization (WHO), were considered to be caused by severe acute respiratory syndrome coronavirus (SARS-CoV-2) from bats (1–3). Since the outbreak, the virus has rapidly spread from Wuhan to China's other areas. As of February 20, 2020, the cumulative number of confirmed cases had reached 66,577 in China, with 2,239 deaths (mortality rate of 3.0%), including 62,442 confirmed cases and 2,144 deaths in Hubei Province. Early observation of infections of health-care workers as well as family members has suggested that human-to-human transmission has occurred through close contacts (4, 5). The epidemic doubled in size every 6.4–7.4 days in its early stage and would lag in imported cities by 1–2 weeks, with the basic reproductive number (*R*_0_) estimated to be 2.2–2.68 (4, 6, 7).

The outbreak of COVID-19 coincided with the eve of the traditional Chinese Spring festival. Many residents visited their relatives and friends, leading to sharply increased transportation, and potential risk of rapid transmission between cities (8). Although Chinese authorities imposed travel bans on Wuhan and several cities near Wuhan since January 23, 2020 (9), it was estimated that ~5 million of residents had left Wuhan before the lockdown, which might contribute to the spread of virus to other domestic cities. After the outbreak, Chinese authorities have taken unprecedented measures to control the source of infection, including screening of high risk populations, prompt identification, and reporting of suspicious cases, and rapid diagnosis of cases (10). The Chinese government required residents to self-quarantine, or stay home from work, and avoid big crowds. By February 20, the number of daily new confirmed cases nationwide had dropped significantly across the country, in particular no new cases for three consecutive days were achieved in some provinces or cities. However, the local epidemiological characteristics of COVID-19 in the imported cities remain unclear. On the other hand, according to the national data, 18.5% of COVID-19 cases in China presented severe symptoms of infections (11). The associations between the severity of disease and epidemiologic factors need investigation.

Tianjin is one of the four municipalities under the direct administration of central government of China. It is located in the northern part of the North China Plain, 1,171 kilometers from Wuhan. Tianjin is one of the representative cities in China, as it has a population of 15.6 million residents, developed economy, and convenient transportation. In this study, we provided an analysis of spatial and temporal distribution of all 131 confirmed cases in Tianjin before February 20, to describe the epidemiologic characteristics of COVID-19. In addition, we reconstructed the transmission chain, and explored the effects of epidemiologic factors on the severity of the disease in this study.

## Methods and Materials

### Data Source

Soon after SARS-CoV-2 was identified as the etiological pathogen of the pneumonia outbreak, the disease was classified as Class B infectious disease and managed as Class A (12, 13). Confirmed patients are required to be reported within 24 h to the National Notifiable Infectious Disease Surveillance System, according to the standard protocol issued by National Health Commission of the People's Republic of China (NHCC). The information of each COVID-19 case was input into the data system by local hospitals and CDC personnel, who investigated and collected possible exposure and exposure route. Each case had a fixed number in the data system in accordance with the order of diagnosis. All case records contained unique personal ID number, so cases were not duplicated in the system.

We collected the COVID-19 epidemic data released from the official website of Tianjin municipal government (http://www.tj.gov.cn/) and the Tianjin Health Committee. The relevant data were collected for analysis after removing all personally identifiable information.

### Variables

Case data included basic demographic information, date of symptom onset, date of isolation, date of medical treatment, date of diagnosis, exposure routes, clinical symptoms, and the severity of disease. Wuhan-related exposure referred to a history that patients recently lived or traveled in Wuhan, or had close contact with a person who had been to Wuhan.

Patients were diagnosed based on clinical symptoms and/or a history of exposure and positive results from viral nucleic acid tests, according to the Diagnosis and Treatment Program of 2019 New Coronavirus Pneumonia issued by the National Health Commission of China (14, 15). Patients were divided into two groups based on the severity of the disease, namely non-severe and severe infection groups. Non-severe infected patients defined as those without pneumonia or mild pneumonia; Severe cases defined as those presented dyspnea, respiratory rate ≥30/min, blood oxygen saturation ≤ 93%, PaO_2_/FiO_2_ ratio <300, and pulmonary infiltration > 50% within 24–48 h; or those cases with respiratory failure, septic shock, and/or multiple organ dysfunction/failure.

The date of onset was defined as the date on which a case began to develop symptoms such as fever or cough according to self-report data in the epidemiological investigation. Date of isolation defined as the date of self-isolation, compulsory isolation, or hospitalization.

### Statistical Analysis

For spatio-temporal analysis, the number of confirmed cases was plotted according to the date of symptom onset and date of diagnosis, respectively. The cumulative numbers of cases before specific time points (January 21, 2020, January 31, 2020, February 10, 2020, and February 20, 2020) were mapped by using ESRI ArcMap 10.4.1 Software according to the geographic location, respectively.

Statistical analysis was performed using SPSS 25.0. Quantitative variables were expressed as means ± standard deviation (*SD*) or medians and percentiles (25th percentile, 75th percentile). Categorical variables were reported as numbers and percentages. The proportions were compared using the chi-squared test. Comparisons of continuous variables between the groups were performed using independent *t*-test for normally distributed data and the Mann-Whitney *U*-test for data not normally distributed. Univariate and multivariate logistic regression analyses were performed to identify risk factors that were associated with severe infection, with results reported as the odds ratio (OR) at 95% confidence interval (CI). *P* < 0.05 was considered statistically significant.

## Results

**Figure 1:**
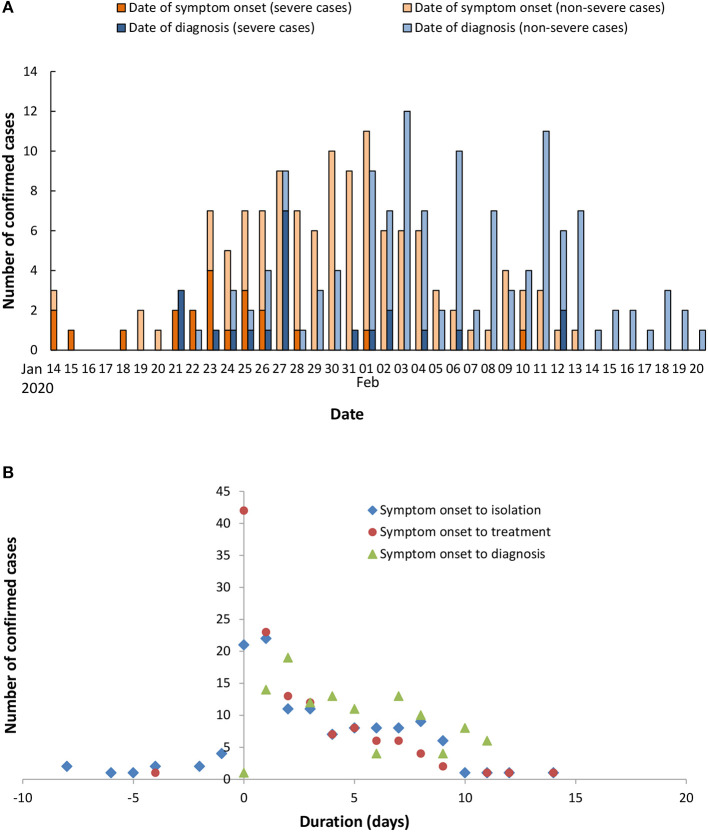
Temporal distribution of confirmed COVID-19 cases in Tianjin. Epidemic curves of confirmed cases were drawn according to the date of onset and the date of diagnosis **(A)**. The distribution of cases was plotted based on specific duration since the onset date of symptoms **(B)**.

**Figure 2:**
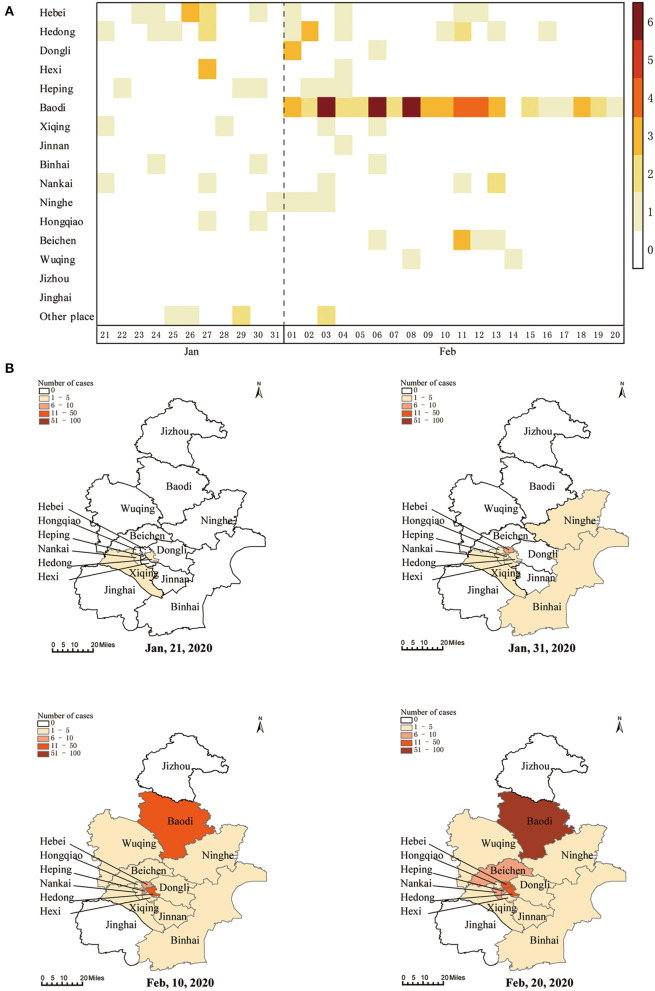
Spatial distributions of confirmed COVID-19 cases in Tianjin. Daily number of new infections in each district **(A)** and cumulative number of cases on map by the end of January 21, 2020, January 31, 2020, February 10, 2020, and February 20, 2020 **(B)**.

A total of 131 COVID-19 cases were diagnosed in Tianjin before February 20, 2020. The first infected patient developed symptom of fever on January 14, 2020, and was subsequently diagnosed on January 21. Epidemic curves of confirmed cases were drawn based on the date of onset and the date of diagnosis (Figure 1A). Also, the numbers of confirmed cases were shown according to the duration of symptom onset to treatment, isolation, and diagnosis (Figure 1B). Geographically, three districts (Hedong, Xiqing, and Nankai) reported confirmed cases at the earliest on January 21 (Figure 2A). By February 20, a total of 14/16 districts reported COVID-19 cases, with Baodi district reporting the most confirmed cases (*n* = 56; Figure 2B).

**Figure 3:**
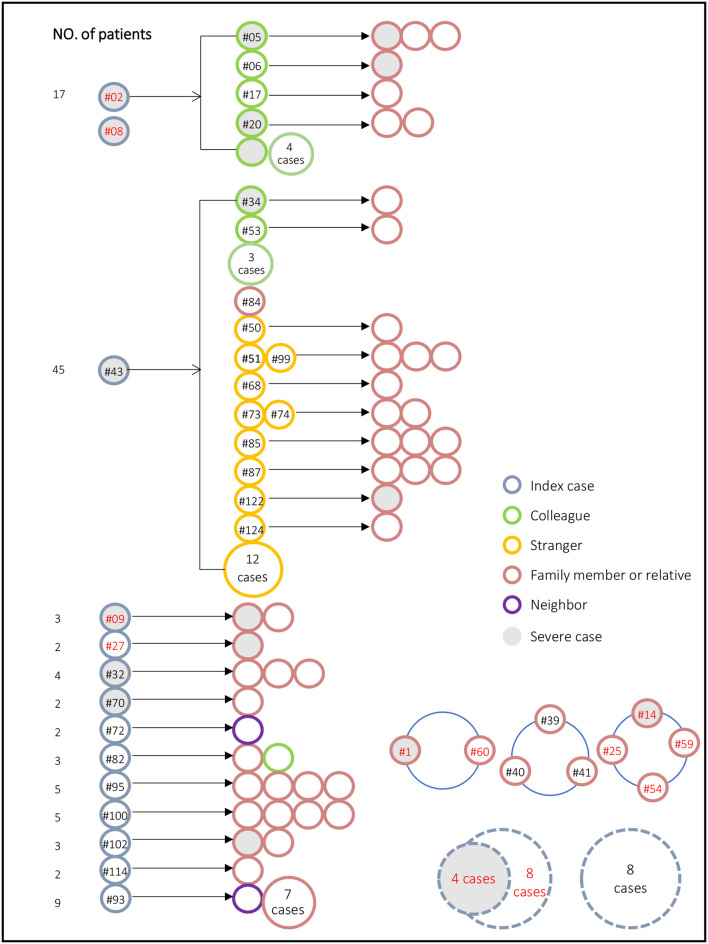
Transmission chain for the 131 confirmed COVID-19 cases in Tianjin. Red figures indicate a Wuhan-related exposure. Gray solid fill represents severe case. Numbers in dashed circle (lower right corner) represent the total number of unrelated independent events, and these patients did not transmit the virus to others. The blue hollow ring in the lower right corner indicates that these patients were infected at the same time or from the same source, for example, #14, #25, #54, and #59 traveled to Wuhan together.

We analyzed the transmission chain of COVID-19 cases in Tianjin (Figure 3). There were two major clusters of infections. In one cluster, two train conductors traveled to Wuhan and developed fever after returning to Tianjin. One of them infected eight of his colleagues, among whom four transmitted the virus to their respective family members. A total of 17 people were infected in the event. The second cluster was from a shopping mall located in Baodi District. A 35-years-old saleswoman with an unclear source of infection developed fever on January 21. Through cross infection, she transmitted the infection to one of her family members, five salespersons and 22 customers. Among them, two salespersons and 10 customers transmitted the virus to their respective family members. The cluster included a total of 45 cases.

The analysis of the transmission chain showed that many index cases had a Wuhan-related exposure (red figures in Figure 3). Transmission from a family member constituted 42%, usually at the end of transmission chain.

Of 131 confirmed cases (male 54.2%), the mean age was 48.7 ± 17.1 years old (Table 1). A total of 22 (16.8%) cases had a Wuhan-related exposure. Fever was the commonest symptom (82.4%). The median duration of symptom onset to treatment was [1.0 (0.0–4.0) days], the duration of symptom onset to isolation [2.0 (0.0–6.0) days], and the duration of symptom onset to diagnosis [5.0 (2.0–8.0) days].

**Table 1:** Characteristics of 131 confirmed cases with COVID-19 in Tianjin.

	**Total**
	**(*n* = 131)**
Age, years	48.7 ± 17.1
Gender
Male	71 (54.2)
Female	60 (45.8)
Wuhan-related exposure
Yes	22 (16.8)
No	109 (83.2)
Source of transmission
Family or relatives	55 (42.0)
Others	76 (58.0)
Fever
Yes	108 (82.4)
No	17 (13.0)
Unclear	6 (4.6)
Cough
Yes	28 (21.4)
No	97 (74.0)
Unclear	6 (4.6)
Fatigue
Yes	11 (8.4)
No	114 (87.0)
Unclear	6 (4.6)
Headache
Yes	12 (9.2)
No	113 (86.2)
Unclear	6 (4.6)

The patients were divided into two groups according to the severity of the disease, non-severe infection (*n* = 93), and severe infection (*n* = 22), which was not included 16 cases whose disease conditions were unclear (Table 2). Compared with patients with non-severe infections, patients with severe infections were more likely to be male (46.2 vs. 77.3%, *P* = 0.009) and had a Wuhan-related exposure (14.0 vs. 40.9%, *P* = 0.004). There was no statistical difference in clinical symptoms including fever, cough, fatigue, and headache between the two groups (*P* > 0.05).

**Table 2:** Characteristic of patients with COVID-19 stratified by disease severity.

**Variables**	**Total (*n* = 115)**	**Disease severity**		***P-value***
		**Non–severe (*n* = 93)**	**Severe (*n* = 22)**	
Age, years	48.2 ± 17.4	47.0 ± 17.8	53.1 ± 15.5	0.145
Age, *n* (%)				
<60 years old	85 (73.9)	69 (74.2)	16 (72.7)	0.888
≥60 years old	30 (26.1)	24 (25.8)	6 (27.3)	
Gender, *n* (%)				0.009
Male	60 (52.2)	43 (46.2)	17 (77.3)	
Female	55 (47.8)	50 (53.8)	5 (22.7)	
Wuhan-related exposure, *n* (%)				0.004
Yes	22 (19.1)	13 (14.0)	9 (40.9)	
No	93 (80.9)	80 (86.0)	13 (59.1)	
Time from onset to initial treatment, days	1.0 (0.0–4.0)	1.0 (0.0–4.0)	1.0 (0.3–3.0)	0.780
Time from onset to isolation, *n* (%)^*^				
≤ 0 days	33 (29.2)	27 (29.4)	6 (28.6)	0.943
≤ 5 days	51 (45.1)	42 (45.6)	9 (42.8)	
>5 days	29 (25.7)	23 (25.0)	6 (28.6)	
Time from onset to diagnosis, days	5.0 (2.0–8.5)	5.0 (2.3–8.0)	4.0 (1.5–9.5)	0.730
Time from onset to diagnosis, *n* (%)^*^				
≤ 5 days	65 (57.5)	54 (58.7)	11 (52.4)	0.579
>5 days	48 (42.5)	38 (41.3)	10 (47.6)	
Source of transmission, *n* (%)				0.080
Family or relatives	45 (39.1)	40 (43.0)	5 (22.7)	
Others	70 (60.9)	53 (57.0)	17 (77.3)	
Fever, *n* (%)				0.284
Yes	95 (82.6)	78 (83.9)	19 (86.4)	
No	16 (13.9)	12 (12.9)	1 (4.5)	
Unclear	4 (3.5)	3 (3.2)	2 (9.1)	
Cough, *n* (%)				0.447
Yes	24 (20.9)	19 (20.4)	5 (22.7)	
No	86 (74.8)	71 (76.4)	15 (68.2)	
Unclear	5 (4.3)	3 (3.2)	2 (9.1)	
Fatigue, *n* (%)				0.346
Yes	11 (9.5)	8 (8.6)	3 (13.6)	
No	99 (86.1)	82 (88.2)	17 (77.3)	
Unclear	5 (4.4)	3 (3.2)	2 (9.1)	
Headache, *n* (%)				0.378
Yes	10 (8.7)	9 (9.7)	1 (4.5)	
No	100 (87.0)	81 (87.1)	19 (86.4)	
Unclear	5 (4.3)	3 (3.2)	2 (9.1)	

* *n = 2 missing data*.

We performed a univariate logistic regression analysis and found that male (OR 3.953, 95% CI 1.346, 11.610; *P* = 0.012) and Wuhan-related exposure (OR 4.260, 95% CI 1.517, 11.962; *P* = 0.006) were risk factors for severe infection (Table 3). Multivariate logistic regression showed that only male (OR 3.913, 95% CI 1.206, 12.696; *P* = 0.023) was an independent risk factor for severe infection.

**Table 3:** Epidemiological factors associated with severe COVID-19 in Tianjin.

**Variables**	**Univariate logistic regression**		**Multivariate logistic regression**	
	**Crude OR (95% CI)**	***P-value***	Adjusted OR^*^ (95% CI)	***P-value***
Age ≥60 years old	1.078 (0.378, 3.071)	0.888	1.682 (0.524, 5.395)	0.382
Male	3.953 (1.346, 11.610)	0.012	3.913 (1.206, 12.696)	0.023
Wuhan-related exposure	4.260 (1.517, 11.962)	0.006	2.294 (0.670, 7.859)	0.186
Transmission from family or relatives	0.390 (0.133, 1.146)	0.087	0.419 (0.115, 1.526)	0.187
Fever	2.923 (0.358, 23.887)	0.317		
Cough	1.246 (0.402, 3.862)	0.704		
Fatigue	1.809 (0.435, 7.528)	0.415		
Headache	0.474 (0.057, 3.968)	0.491		

* *ORs were adjusted for age, gender, Wuhan-related exposure and source of transmission*.

## Discussion

In this study, we retrospectively analyzed 131 confirmed COVID-19 cases in Tianjin, and the results showed that SARS-CoV-2 infection occurred in 14/16 districts, with the most cases in Baodi district. Transmission from a family member constituted 42%, usually at the end of transmission chain. Although SARS-CoV-2 was highly contagious, most patients had mild manifestations. Male was a risk factor for severe infection.

First, we displayed the epidemiological characteristics of confirmed cases of COVID-19 in Tianjin. Tianjin lies between 116°43' to 118°04' e and 38°34' to 40°15' n. The city has 16 districts with a total area of 11,966 km^2^ and a population of 15.6 million. The epidemic curve showed that 51.2% (65/127) of patients were treated or seek medical treatment on the day or next day of symptom onset (0–1 day). A small number of patients (9.4%, 12/127) who had close contact with an infected individual were isolated before symptoms appeared. The period from symptom onset to diagnosis was 5.0 (2.0–8.0) days.

Another contribution of this study is to describe the transmission chain and spreading pattern of SARS-CoV-2 in Tianjin. Since January 23, Chinese government required individuals with Wuhan-related exposure history to report personal information and quarantine themselves, regardless of infection. In this study, a total of 22 (16.8%) cases had a Wuhan-related exposure. Except for the initial cases, most of these patients implemented self-quarantine after the outbreak, thus they did not infect other people, suggesting the importance of self-quarantine especially for those with high-risk exposures. In addition, Chinese authorities required residents to stay at home, avoiding outdoor activities. As a result, a considerable proportion of transmissions occurred between families or relatives; however, this broke the chain of infection transmission and therefore prevented the spread of COVID-19. On the contrary, outside activities, such as transmission in the shopping mall at Baodi district, led to complex cross transmission and a wider range of transmission. This is consistent with the findings from Kim et al. who found that transmission of Middle East respiratory syndrome coronavirus was determined by the number of contacts (16). Therefore, it is crucial to isolate patients and trace and quarantine contacts as early as possible. The data from transmission chain analysis will help to make decision for some regions that have not yet begun or are experiencing a COVID-19 epidemic.

The SARS-CoV-2 is mainly transmitted through respiratory droplets. After infection, patients may show bilateral ground-glass opacity or consolidation on chest CT scans, along with common symptoms that include fever, dry cough, and shortness of breath, at the onset of illness (17–19). In severe cases, dyspnea, respiratory distress syndrome, or septic shock may develop (18). In this study, 19.1% of cases presented severe manifestations. We analyzed the risk factors for early severe infections in Tianjin. In a recently published study with a sample size of 72,314, the mortality rate for men was significantly higher than that for women (63.8 vs. 36.2%) (11). Our study found that male was an independent risk factor for severe infection, suggesting the necessity of paying more attention to early intervention. We speculated on the causes of the association between male and severe COVID-19 infection. Males and females differ in their immunological responses to pathogens, and males generally show higher susceptibility, prevalence, and severity of infection than females, including respiratory tract infection (20–22). Observed in mice infected with *Mycoplasma pulmonis*, the pulmonary parenchyma disease of male mice is always more serious than that of female mice (23). On the other hand, smoking is generally more prevalent among men than women. Although no firm conclusions can be drawn about the association between smoking and severity of COVID-19 (24), some evidence shows that active cigarette smoking and up-regulation of ACE-2 expression (an entry receptor of SARS-CoV-2) in lower airways may in part contribute to the increased risk of severe COVID-19 (25). Further investigation is needed to confirm the association between male and severe COVID-19 infection and investigate the accurate mechanisms. The severe infection group had a higher proportion of patients with Wuhan-related exposure, which, however, was not an independent risk factor. In fact, most of these patients were infected in the early stage of the epidemic, when the disease might be easily ignored or diagnosed delayed. Based on a previous study, patients treated in the intensive care unit (ICU) (*n* = 36), compared with patients not treated in the ICU (*n* = 102), were older (median age, 66 vs. 51 years) (26). Also, another study showed that older age were associated with severe infection (27). In our study, the mean age of patients with severe infections was higher than that of patients with non-severe infections, but the difference was not statistically significant (*P* = 0.145). Further studies with larger sample size are needed for determining whether age is a risk factor for severe infection.

This study provided detailed data regarding the COVID-19 epidemic of Tianjin, a representative city in China. However, this study has some limitations. First, although we collected all confirmed cases before February 20, 2020 in Tianjin for analysis, the sample size was still small. Secondly, all cases in this study were clinically diagnosed, and a fairly high percentage of cases were investigated by professional epidemiologists. However, some data were not be collected or missed in the system, such as underlying diseases or comorbidities. Thirdly, memory bias might exist in the epidemiological investigation, for instance, date of symptom onset, which could lead to inaccurate estimates of some variables. In addition, the proportion of patients with cough in this study (21.4%) was lower than the two earlier reports (67.7 and 59.4%) (26, 28). It might be caused by a self-reported data of patients on early symptoms.

In conclusion, this study provides important information on the epidemic of COVID-19 by analyzing the epidemiological characteristics of confirmed cases in Tianjin. We suggest that self-quarantine at an outbreak's early stage, especially for those with high-risk exposures, is conducive to prevent the transmission of infection. Further investigation is needed to confirm the risk factors for severe COVID-19 infection and investigate the mechanisms involved.

## Data Availability Statement

The raw data supporting the conclusions of this article will be made available by the authors, without undue reservation, to any qualified researcher.

## Ethics Statement

This study was approved by the Human Subjects Review Board of the School of Public health, Sun Yat-sen University [No. 2020 (003)]. Informed consent was obtained from all individual participants included in the study.

## Author Contributions

JL and ZC designed the study. JW, XC, HH, ZL, CL, and PL conducted the collection of the data. ZL, XC, and HH performed the statistical analysis. JW and ZC interpreted the results and wrote the first draft of the manuscript. JL corrected the manuscript. All authors contributed to manuscript, and approved the submitted version.

## Conflict of Interest

The authors declare that the research was conducted in the absence of any commercial or financial relationships that could be construed as a potential conflict of interest.
